# Distributed Event-Driven Serverless Platform for Multicluster IoT Environments

**DOI:** 10.3390/s26051718

**Published:** 2026-03-09

**Authors:** Hyungwoo Ju, Jangwon Seo, Younghan Kim

**Affiliations:** School of Electronic Engineering, Soongsil University, Seoul 06978, Republic of Korea; wnguddn1234@dcn.ssu.ac.kr (H.J.); sjw98052323@dcn.ssu.ac.kr (J.S.)

**Keywords:** serverless architecture, event-driven system, IoT systems, real-time processing, multi-event

## Abstract

In modern smart city and IoT environments, diverse sensors for traffic management, environmental monitoring, and energy systems continuously generate large volumes of heterogeneous events in real time. Efficiently processing these multi-source event streams requires a scalable and responsive computing architecture. However, many Kubernetes-hosted serverless Function-as-a-Service (FaaS) deployments operate within a single administrative cluster and provide limited user-level control over dynamic multicluster placement based on heterogeneous event types and real-time resource conditions. To address these limitations, this study proposes a generalized event-driven FaaS architecture capable of efficiently processing multi-event streams across multicluster environments. The proposed architecture was implemented on Kubernetes-based testbed by integrating a multicluster orchestrator, an event-processing engine, a workflow execution layer, and a serverless platform. Evaluation using a smart city-inspired scenario demonstrates that the proposed platform provides improved load distribution characteristics and maintains higher workflow success rates under increasing workloads compared to the evaluated single-cluster baseline. This research provides a scalable design approach for serverless platforms that can meet real-time event processing requirements in IoT and smart city applications.

## 1. Introduction

In modern smart city and Internet of Things (IoT) environments, large volumes of heterogeneous and continuous events are generated in real time from diverse sources such as traffic monitoring systems, environmental sensors, energy management devices, and safety surveillance applications. These events go beyond simple data collection and serve as key enablers for real-time analytics and immediate responses that improve urban operational efficiency and service quality. In such environments, the importance of computing systems capable of processing large-scale heterogeneous events with low latency and flexibly adapting to dynamic and fluctuating workloads has significantly increased. To address these requirements, serverless and event-driven computing models have emerged as core technologies in IoT systems [1,2,3]. Serverless computing enables efficient resource utilization through on-demand resource allocation, while event-driven architectures facilitate the automation of complex processing logic by leveraging diverse sensor events as execution triggers [4].

However, many existing Kubernetes-based event-processing and serverless platforms are primarily designed for single-cluster deployments, where multicluster coordination and placement control require additional federation layers or manual configuration [5,6]. First, processing all event types and volumes within a single cluster causes severe resource bottlenecks and limits scalability as the number and heterogeneity of IoT events grow. Second, complex workloads such as AI inference and large-scale data pipelines can overload a single cluster, eventually causing resource exhaustion, performance degradation, and event loss under high demand scenarios [7]. Third, binding event-processing logic and function execution to a single cluster restricts the ability to leverage geographically distributed edge cloud resources. Furthermore, when extending to multicluster environments, cluster federation, coordination, and policy management often require extensive manual configuration, significantly increasing operational complexity [8,9]. These challenges highlight the need for an intelligent, distributed event-driven orchestration platform that can dynamically schedule events and functions across multiple clusters while reflecting real-time resource conditions.

Prior research has explored individual aspects such as multicluster execution, serverless scalability, and event-processing performance. However, few studies have proposed a comprehensive architecture that integrates diverse IoT event streams and executes them seamlessly across distributed clusters. To address this gap, this paper introduces a generalized event-driven Function-as-a-Service (FaaS) architecture designed to efficiently process real-time IoT events in multicluster environments. The proposed architecture consists of four core layers: (1) an event-workflow orchestration layer that generates event, workflow, and function resources based on user-defined specifications; (2) a resource-aware placement engine that determines execution locations by considering real-time cluster resource states; (3) a distributed event bus that collects and routes events to selected clusters; and (4) a serverless function execution layer that executes event-triggered functions with automated scaling. Together, these components enable unified orchestration, dynamic placement, and distributed execution of heterogeneous IoT events across multiple clusters [10]. From a conceptual perspective, our work differs from conventional distributed event-based middleware and event engines widely used in IoT (e.g., publish/subscribe brokers and stream/CEP engines) in that it treats event handling and computation placement as a unified orchestration problem, while existing IoT event engines mainly focus on event routing, filtering, and detection within a fixed execution domain, the proposed platform explicitly integrates (i) multi-event condition evaluation, (ii) workflow-driven execution, and (iii) serverless function scaling with (iv) resource-aware multicluster placement. This integration enables IoT operators to benefit from FaaS characteristics (on-demand scaling and resource efficiency) while maintaining stable event processing under bursty workloads and cluster-level resource contention. Therefore, the primary contribution of this paper is architectural integration and automated multicluster orchestration, rather than proposing a new event processing engine or a novel placement algorithm.

To validate the proposed architecture, we implemented the system in a Kubernetes-based multicluster environment and conducted experiments using realistic smart city scenarios. Experimental results demonstrate that the proposed platform provides stable latency characteristics while improving load distribution and maintaining higher workflow success rates under increasing workloads compared with the evaluated single-cluster baseline. This study presents a scalable serverless architecture capable of meeting the growing demands of large-scale, real-time IoT event processing in smart city environments and other large-scale IoT deployments.

## 2. Related Work

In modern IoT and smart city environments, large volumes of complex and heterogeneous events are continuously generated from sensors, cameras, autonomous vehicles, and edge devices. Scalable computing architectures capable of processing these events in real time have therefore become essential. Recent research trends can largely be categorized into event-driven processing models, serverless orchestration frameworks, and multicluster resource management approaches. This section reviews existing studies in these three domains.

### 2.1. Event-Driven Processing in IoT and Smart City Systems

Events generated by IoT sensors must be processed in real time, as processing delays directly affect the stability and reliability of the overall service. Prior studies have proposed event stream processing, complex event detection, and event-driven decision making techniques that focus on rapidly collecting, filtering, and analyzing high volume event data [11,12]. However, most existing approaches rely on a single data center or a single edge node, making them insufficient for distributed IoT infrastructures, where large scale multi-source events are generated across geographically dispersed regions [13]. Consequently, prior work does not provide sufficient architectural support for scalable and distributed event processing [14].

### 2.2. Serverless and Workflow-Based Orchestration

Serverless computing improves resource efficiency by executing functions only when triggered by events. Existing research has explored reducing latency in serverless environments, optimizing function performance, and designing workflow-based architectures to automate complex event processing pipelines [15]. Workflow-driven serverless models have also been proposed to integrate data pipelines and AI inference tasks, enabling multi-step event-triggered computation [16,17].

Nevertheless, these approaches generally assume a single-cluster execution model, which limits their applicability to highly dynamic IoT systems. These approaches fall short in supporting diverse event types, bursty sensor workloads, or dynamic service migration across distributed environments. In particular, complex event patterns (e.g., AND/OR conditions), multi-stage workflows, and AI-assisted processing distributed across multiple clusters are not sufficiently addressed in prior research [18].

**Figure 1:**
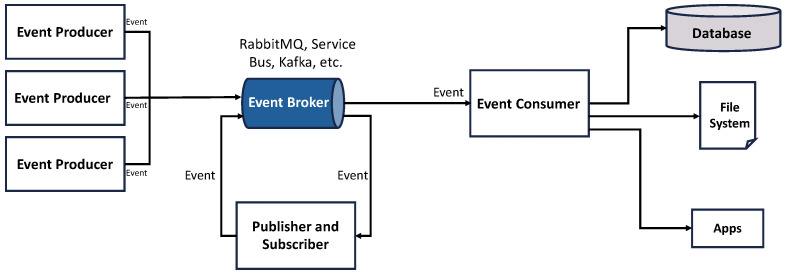
Typical architecture of an event-driven system consisting of event producers, event brokers, publishers/subscribers, and event consumers.

Figure 1 illustrates the typical architecture of an event-driven system composed of event producers, brokers, publishers/subscribers, and event consumers.

### 2.3. Multicluster Orchestration and Resource Placement

Multicluster environments are increasingly important in smart city, autonomous driving, and AI-driven applications, where edge and cloud resources distributed across different locations must be managed as a unified logical computing environment. Previous studies have proposed cluster-aware workload placement policies, policy-based resource propagation mechanisms, and scheduling algorithms driven by real-time resource metrics [19]. Research has also explored unified API management across clusters, federation-based resource control, and cross-cluster data synchronization [20].

However, most prior work limits placement granularity to Deployments or Pods, without considering the entire IoT event processing pipeline, which includes event ingestion, condition evaluation, workflow execution, and serverless function invocation. Furthermore, real-time placement decisions based on fluctuating resource usage, directional event routing between clusters, and distributed event-bus-based multicluster event delivery remain open challenges in existing systems [21].

## 3. Design Architecture

This paper proposes an event-driven Function-as-a-Service (FaaS) platform that receives heterogeneous events across multiple clusters and automatically places function execution resources by reflecting the real-time resource conditions of each cluster. The proposed system integrates Argo Events, Argo Workflows, and Knative Serving on top of a Karmada-based multicluster management framework, enabling full automation from event generation to function execution.

The system consists of a management cluster and multiple member clusters. The management cluster hosts the Orchestrator Controller, which manages end-to-end orchestration, and the Placement Decision Controller, which selects the optimal cluster for execution based on resource metrics. Each member cluster is pre-configured with the necessary components for event processing and function execution, allowing distributed and automated operation across the multicluster environment.

**Figure 2:**
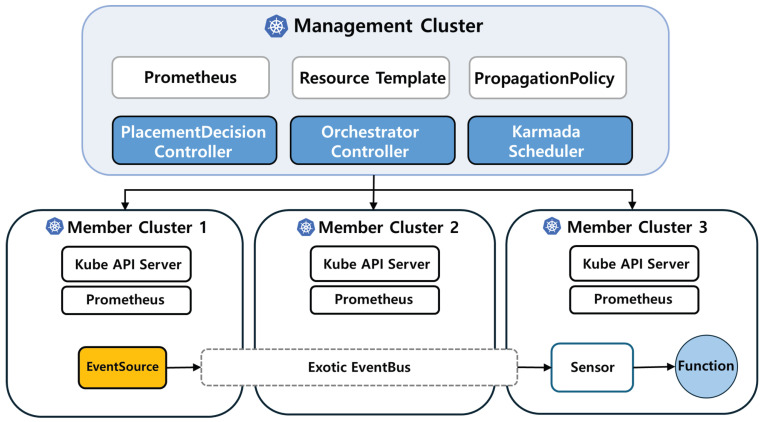
Architecture of an event-driven Function-as-a-Service (FaaS) platform in a multicluster environment.

Figure 2 illustrates the overall architecture of the proposed system. When a user creates an Orchestrator Custom Resource, the Orchestrator Controller detects it and automatically generates the required event-processing resources (EventSource, Sensor, WorkflowTemplate, and Knative Service) as well as policy resources (PropagationPolicy and OverridePolicy).

In parallel, the Placement Decision Controller periodically collects monitoring metrics such as CPU and memory usage from Prometheus for each cluster. These metrics are then scored to determine the most suitable cluster, and the result is recorded in the status field of the PlacementDecision resource. Once updated, the Orchestrator Controller reflects this placement result in the ClusterAffinity field of the corresponding PropagationPolicy, enabling all related resources to be deployed to the selected cluster.

Through this mechanism, users can automate end-to-end deployment of event-driven services across multiple clusters using only a single Custom Resource definition, without manually creating complex policies or writing additional YAML configurations.

**Figure 3:**
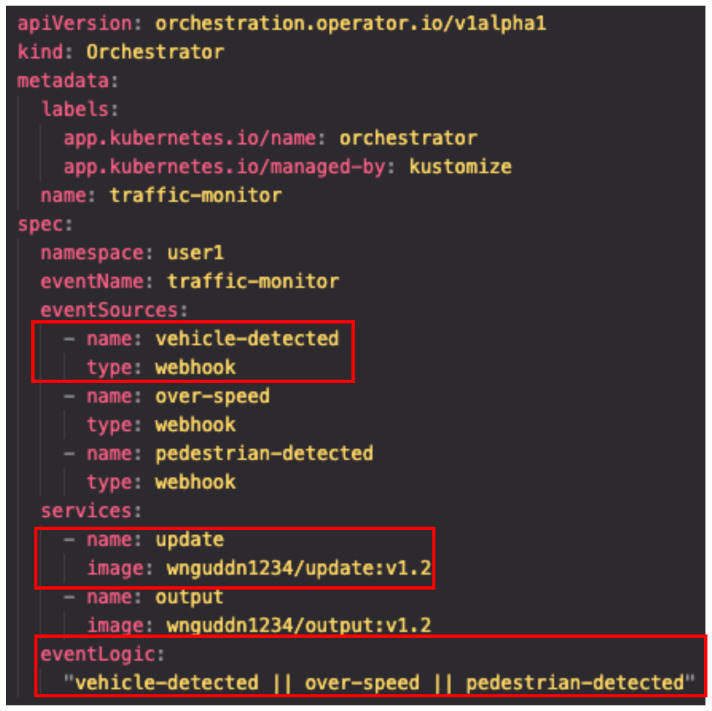
Example of a user-defined Orchestrator custom resource specifying event sources, services, and event logic.

Figure 3 shows the user-defined structure of the proposed system. The Orchestrator Custom Resource (CR) is a high-level abstraction specified by the user and contains only minimal information, such as event logic, service images, and the target deployment namespace. Its main fields include the names of the events to be used, the namespace in which resources will be deployed, the types of event sources, the event-processing logic (e.g., AND/OR conditions), and the container images to be executed. Based on these fields, the Orchestrator Controller automatically generates the following resources:

- EventSource: Creates endpoints for receiving the specified event sources.
- Sensor: Subscribes to events from the EventSource and triggers a workflow when the configured conditions are satisfied.
- WorkflowTemplate: Defines function execution using Argo Workflows (e.g., HTTP calls).
- Knative Service: Exposes the function execution result as a service.

Figure 3 illustrates an example of a user-defined Orchestrator custom resource specifying event sources, services, and event logic.

The Placement Decision Controller is a key component that determines the optimal deployment cluster by continuously collecting cluster resource states from monitoring tools. It operates through the following steps:

1. Metric Collection (Monitoring): Collects CPU and memory usage for each cluster via the Prometheus API.
2. Normalization: Normalizes the collected metrics to a range between 0 and 1.
3. Scoring: Computes a score for each cluster based on weighted normalized resource metrics.

The cluster score is computed as a weighted sum of normalized resource metrics as follows:

(1) $$Score_{i}=w_{cpu}\cdot {C\hat{P}U}_{i}+w_{mem}\cdot {M\hat{e}m}_{i}$$

where ${C\hat{P}U}_{i}$ and ${M\hat{e}m}_{i}$ denote the normalized CPU and memory utilization of cluster *i*, respectively.

In our experimental evaluation, equal weights were used for CPU and memory utilization, i.e., $w_{cpu}=0.5$ and $w_{mem}=0.5$, to avoid bias toward a specific resource and to focus on validating the effectiveness of the multicluster orchestration mechanism. These weights were fixed across all experiments to ensure reproducibility and fair comparison between scenarios.

**Figure 4:**
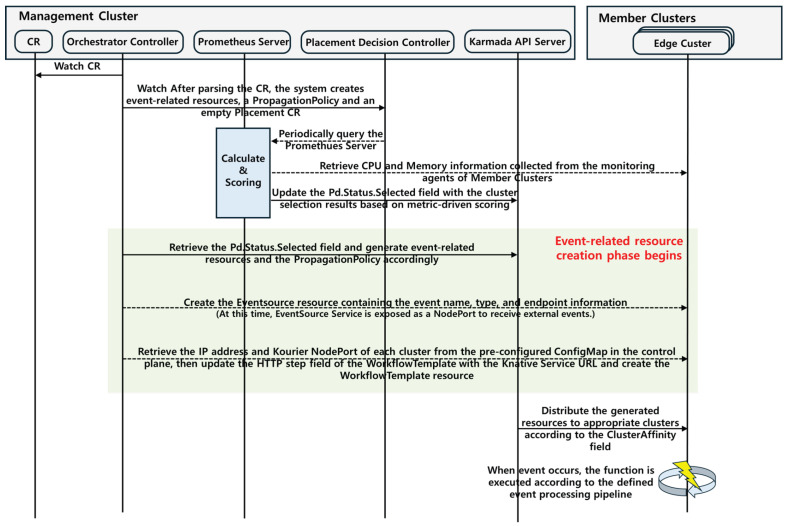
Detailed workflow of event pipeline generation, showing how event sources, sensors, workflows, and serverless functions are automatically created and deployed across clusters.

Figure 4 illustrates the operational workflow of the proposed system. When the user creates a Custom Resource (CR), the Orchestrator Controller detects it and automatically generates the required event-processing resources (EventSource, WorkflowTemplate, and Knative Service), along with the PropagationPolicy for distributed deployment and an empty Placement CR. The Placement Decision Controller periodically collects CPU and memory metrics from each cluster through the Prometheus server, performs normalization and scoring, and updates the most suitable cluster in the PlacementDecision.status.selected field.

The Orchestrator Controller then reads this field and updates the PropagationPolicy so that the WorkflowTemplate, EventSource, Sensor, and other resources are deployed to the selected cluster. The IP addresses of each cluster and the Kourier NodePort values are retrieved from a preconfigured ConfigMap, enabling the system to dynamically configure the Knative Service URL in the WorkflowTemplate’s HTTP step.

Once an EventSource receives an external event, the Sensor evaluates the event conditions and, upon satisfaction, triggers the WorkflowTemplate, leading to function execution through Knative Service. Additionally, when cluster resource conditions change and the PlacementDecision result is updated, the pipeline resources are automatically redeployed to maintain service continuity and ensure effective load balancing across clusters.

**Figure 5:**
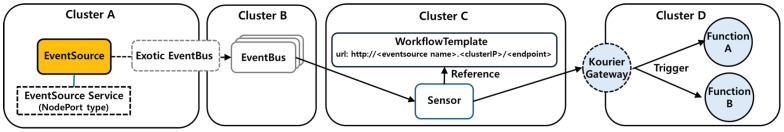
End-to-end event processing workflow across multiple clusters, showing how an event is generated, forwarded through the EventBus, evaluated by the Sensor, and ultimately triggers serverless functions in the target cluster.

Figure 5 illustrates the end-to-end event-processing pipeline in the proposed system, from event generation to function execution. The event-processing workflow is carried out through the interaction of the Exotic EventBus, Sensor, WorkflowTemplate, and Knative Service deployed across multiple clusters. When an event occurs, the EventSource in Cluster A receives it. The EventSource accepts external requests through a NodePort-based EventSource Service and forwards the event data across clusters using the Exotic EventBus.

The Exotic EventBus is a logical component defined in this study, implemented on top of NATS JetStream, to enable cross-cluster event dissemination and external access in a multicluster environment. The term “Exotic” is used to emphasize that the EventBus is configured beyond a conventional single-cluster setup, supporting multicluster subscription, external connectivity, and event sharing across geographically distributed clusters. As a result, events are not restricted to a single processing path and they may be received and processed simultaneously in multiple clusters, such as Cluster A and Cluster C. This behavior is enabled by the PropagationPolicy, which distributes event-related resources EventSource, Sensor, WorkflowTemplate, and others across several clusters. Consequently, the system can process events in parallel across clusters, reducing load on any single cluster and ensuring continued event processing even in the presence of failures.

Upon receiving an event, the Sensor evaluates it against the predefined EventLogic conditions. If the conditions are satisfied, the Sensor triggers the corresponding WorkflowTemplate. Each WorkflowTemplate contains a target service URL in the following format: http://<eventSource-name>.<ClusterIP>/<endpoint>, and the triggered request is delivered through the Kourier Gateway to the Knative Service.

Through this architecture, the proposed system supports simultaneous event reception across multiple clusters and enables distributed event processing, achieving both high resource efficiency and resilience against event loss.

## 4. Experimental Implementation

### 4.1. Testbed Environment

The proposed system was implemented in a multicluster environment consisting of one management cluster and three member clusters (Table 1). Each cluster was deployed on OpenStack-based virtual machine instances (m1.large, 4 vCPUs, 8 GB RAM), and all nodes operated on Ubuntu 20.04 LTS.

### 4.2. Implementation Details of Core Components

#### 4.2.1. Event Processing with Argo Events

In the proposed system, Argo Events is responsible for ingesting external events and evaluating event conditions [22]. These event-handling components—EventSource and Sensor—are automatically generated by the Orchestrator Controller based on the user-defined Orchestrator Custom Resource (CR).

- EventSource is automatically configured to receive external HTTP events through a NodePort exposed EventSource Service.
- The incoming event data is forwarded to an Exotic EventBus, implemented using NATS JetStream, enabling multiple member clusters to simultaneously subscribe to the same event stream.
- Sensor resources are dynamically created according to the EventLogic specified in the Orchestrator CR (e.g., AND/OR conditions and filtering rules). When the specified conditions are satisfied, the Sensor triggers the corresponding WorkflowTemplate.

**Figure 6:**
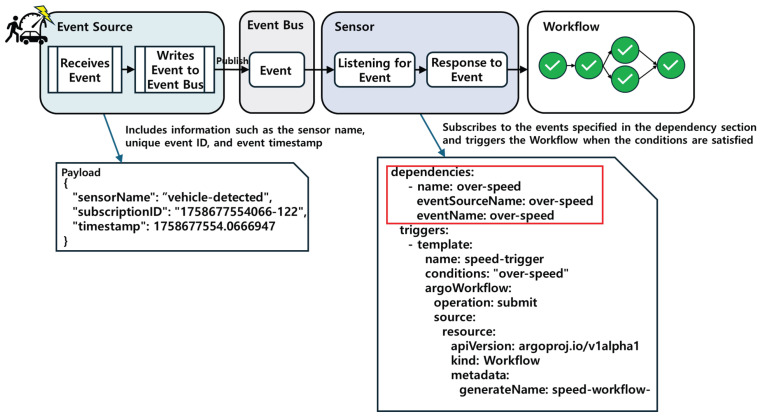
Basic event-processing pipeline showing how an event is received by an EventSource, published to the EventBus, evaluated by a Sensor, and finally triggers the corresponding function.

Figure 6 illustrates the basic event-processing pipeline, showing how an event is received by an EventSource, published to the EventBus, evaluated by a Sensor, and finally triggers the corresponding function.

#### 4.2.2. Workflow Execution Pipeline Using Argo Workflows

Argo Workflows provides the function execution logic in the form of WorkflowTemplates, which are triggered by events [23]. In the proposed system, the Orchestrator Controller automatically constructs these templates as follows:

- The HTTP step of each WorkflowTemplate is dynamically generated to include the Knative Service URL of the selected cluster.
- The cluster selected by the Placement Decision Controller is recorded in the status field of the PlacementDecision object, which the Orchestrator Controller references to update the WorkflowTemplate accordingly.
- Whenever the placement decision changes, the corresponding WorkflowTemplate is automatically rewritten and redeployed.

This mechanism ensures that workflow execution is always placed in the most optimal cluster based on real-time resource conditions.

#### 4.2.3. Serverless Function Execution Environment with Knative Serving

Knative Serving provides the serverless execution environment for functions triggered after event processing [24]. In the proposed system, Knative is utilized as follows:

- The NodePort information of the Kourier Gateway is stored in a ConfigMap, and the controller automatically injects the corresponding endpoint into the WorkflowTemplate URL.
- Through the scale-to-zero mechanism, Knative terminates function Pods when no events are present and recreates them on demand when events occur.
- In multicluster environments, identical functions can be deployed in parallel across clusters through integration with PropagationPolicy.

This architecture minimizes function execution costs while maintaining rapid response times.

#### 4.2.4. Multicluster Deployment and Resource Management with Karmada

Karmada is responsible for multicluster resource propagation and policy-based placement in the proposed system [25].

- The Orchestrator Controller automatically generates PropagationPolicy and OverridePolicy objects corresponding to EventSource, WorkflowTemplate, and Knative Service resources.
- The cluster selected by the Placement Decision Controller is automatically reflected in the ClusterAffinity field of the PropagationPolicy.
- When placement policies change, Karmada migrates the target resources to the newly selected cluster and removes them from the previous one.

Through this mechanism, the system supports multicluster load balancing, fault tolerance, and automated policy-driven resource deployment.

## 5. Results and Discussions

### 5.1. Experimental Environment

The experiments were conducted by emulating a smart city traffic scenario, where vehicle-detection, pedestrian-detection, and over-speed sensors were configured as EventSources. Each sensor generated event streams through external HTTP (webhook) requests. The experimental system consisted of one Management Cluster and three Member Clusters. All clusters were deployed on OpenStack-based virtual machine instances (m1.large, 4 vCPU, 8 GB RAM) running Ubuntu 20.04 LTS. Each Member Cluster was preconfigured with Argo Events, Argo Workflows, and Knative Serving, while the Management Cluster hosted the Orchestrator Controller and the Placement Decision Controller for system-wide orchestration and resource-aware placement.

The workload was generated via controlled HTTP webhook injections to emulate a smart-city-inspired event scenario, rather than being derived from a real-world smart city dataset. Therefore, the results should be interpreted as an experimental evaluation that demonstrates scalability trends and system behavior under increasing event loads.

### 5.2. Experimental Scenarios

To evaluate the distributed processing performance and stability of the proposed system, two experimental scenarios were designed:

- Scenario A: Single-Cluster Event Processing

In this configuration, all EventSource, Sensor, WorkflowTemplate, and Knative Service resources are deployed in a single cluster. This setup reflects the conventional architecture used in existing serverless and event-driven frameworks, where all pipeline components operate within one cluster.

- Scenario B: Multicluster Dynamic Distributed Placement

In this scenario, the Placement Decision Controller selects the most appropriate cluster based on Prometheus metrics (e.g., CPU and memory usage). Karmada then automatically propagates and deploys the pipeline components EventSource, Sensor, WorkflowTemplate, and Knative Service across clusters according to the placement decision. For both scenarios, each sensor (EventSource) generated between 30 and 100 simultaneous Webhook requests. The full lifecycle of event ingestion and function execution was measured, enabling comparative analysis of processing performance and system stability between the single-cluster baseline and the proposed multicluster distributed placement.

**Figure 7:**
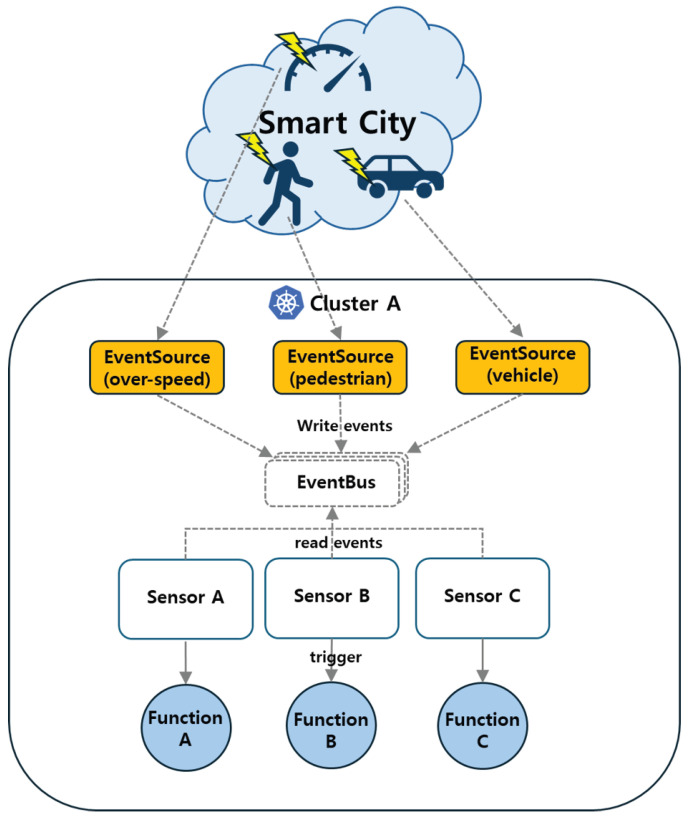
Single-cluster event processing pipeline, where multiple EventSources publish events to a shared EventBus, which then triggers functions within the same cluster.

Figure 7 illustrates the single-cluster event processing pipeline, where multiple EventSources publish events to a shared EventBus, which then triggers functions within the same cluster.

**Figure 8:**
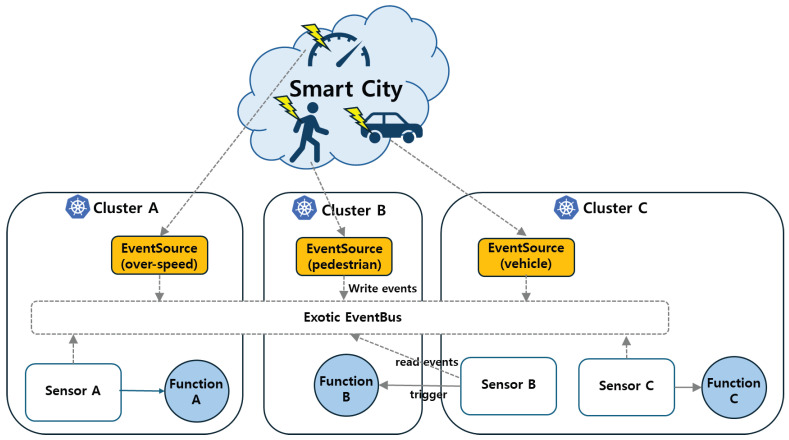
Multicluster event processing pipeline, where EventSources publish events locally while the EventBus forwards them to functions deployed in another cluster.

Figure 8 illustrates the multicluster event processing pipeline, where EventSources publish events locally while the EventBus forwards them to functions deployed in another cluster.

### 5.3. Performance Metrics

To evaluate the performance and stability of the proposed system, we measured the total end-to-end latency from event reception to function invocation, as well as the workflow success rate, failure rate, and pending rate for each event request *r*. The total processing time from event arrival to function execution is calculated as follows:

(2) $$T_{TTF}\left(e\right)=T_{es}+T_{bus}+T_{ss}+T_{f}$$

- $T_{TTF}\left(e\right)$: Total processing time from event arrival to function execution.
- $T_{es}$: Time for the event to be received by the EventSource.
- $T_{bus}$: The time taken for the event to be published to the EventBus.
- $T_{ss}$: The time required for the event to be delivered from the EventBus to the Sensor.
- $T_{f}$: The time from the Sensor triggering the workflow until the function is invoked.

**Figure 9:**
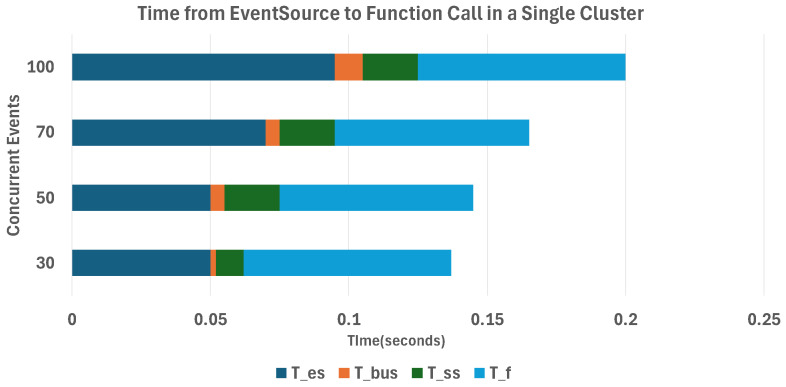
Breakdown of end-to-end latency from EventSource to function invocation in a single-cluster environment.

Figure 9 illustrates the breakdown of end-to-end latency from the EventSource to function invocation in a single-cluster environment.

**Figure 10:**
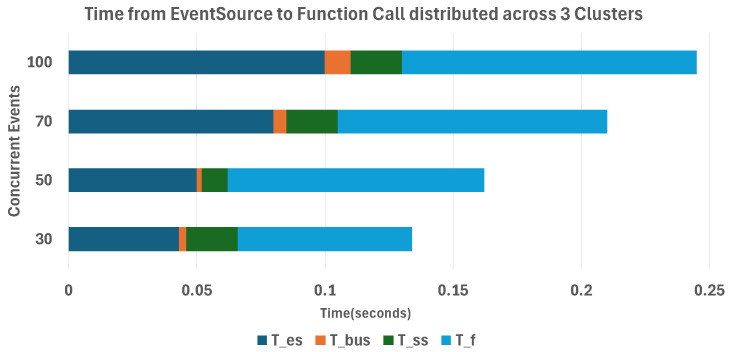
Latency breakdown from EventSource to function invocation in a three-cluster environment (30–100 concurrent events).

Figure 10 illustrates the latency breakdown from the EventSource to function invocation in a three-cluster environment (30–100 concurrent events).

The experimental results show that in the single-cluster environment, the average end-to-end latency was approximately 0.18 s, benefiting from fewer network hops. In contrast, the multicluster environment exhibited an average latency of 0.27 s, representing roughly a 1.5x increase. This additional overhead is primarily attributed to inter-cluster communication, where requests must traverse the Ingress Gateway before reaching the target cluster.

Although the average latency in the multicluster case increased to 0.27 s, the p95 latency remained below 0.35 s across all workloads, indicating stable performance under distributed execution. Previous studies have reported that multicluster and geo-distributed environments inherently introduce tens to hundreds of milliseconds of latency due to increased network hops and service mesh routing complexity [13,26,27]. Therefore, in large-scale IoT scenarios where event rates are high and lightweight processing is common, the observed latency overhead is considered negligible and does not impede real-time responsiveness.

Additionally, the workflow success rate, failure rate, and pending rate for each event request *r* were measured to evaluate overall system stability. All experiments were performed under identical hardware specifications across clusters (4 vCPU, 8 GB memory, and 74 GB storage) to ensure a fair performance comparison:

(3) $$N_{total}\left(r\right)=N_{success}+N_{failed}+N_{pending}$$

- $N_{success}$: Number of requests for which the workflow completed successfully.
- $N_{failed}$: Number of requests that failed due to errors during workflow execution.
- $N_{pending}$: Number of requests that remained in a pending state, typically due to insufficient resources preventing Pod creation.

In this study, a workflow is classified as pending when it cannot be scheduled immediately due to temporary resource shortages affecting the Kubernetes scheduler, such as CPU or memory saturation. This pending state reflects resource pressure rather than limitations of the Argo Workflow controller itself. In the multicluster environment, pending workflows may trigger redistribution decisions by the Placement Decision Controller, allowing execution to continue on less congested clusters.

**Figure 11:**
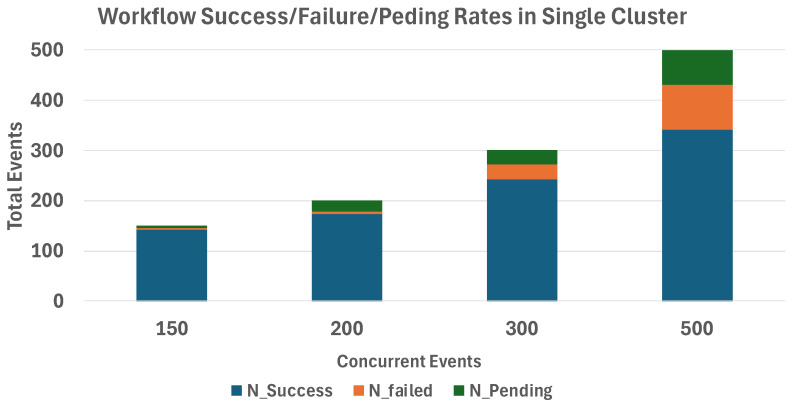
Workflow success, failure, and pending event counts in a single-cluster environment under increasing concurrent event loads.

Figure 11 shows the workflow success, failure, and pending event counts in a single-cluster environment under increasing concurrent event loads.

**Figure 12:**
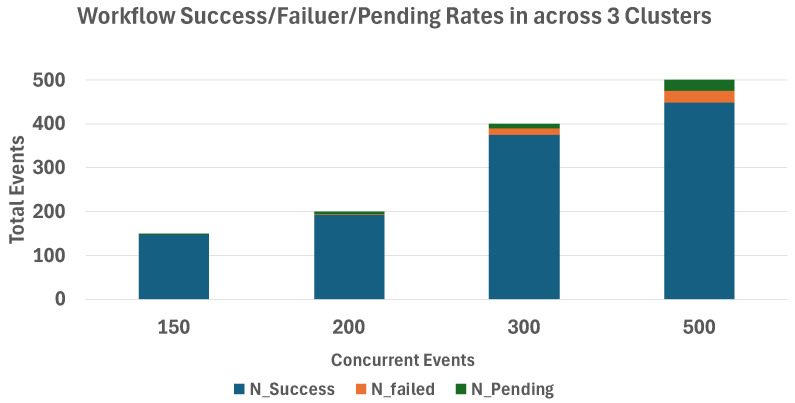
Workflow success, failure, and pending event counts in a three-cluster environment under increasing concurrent event loads.

Figure 12 shows the workflow success, failure, and pending event counts in a three-cluster environment under increasing concurrent event loads.

Using Equation (2), we compute the workflow success rate as $R_{success}=N_{success}/N_{total}$, where $N_{total}$ includes success, failed, and pending outcomes. When 100 concurrent HTTP requests were issued, both environments achieved success rates above 95%. As the number of requests increased to 500, the single-cluster environment showed a decrease in success rate to 84%, whereas the multicluster configuration maintained a success rate of 97%.

We note that increasing the number of clusters also increases the aggregate compute and memory capacity available to the system. Therefore, part of the observed improvement may be attributable to increased overall capacity rather than solely to the placement mechanism itself. In this study, the primary objective is not to establish strict causal isolation of dynamic placement effects, but to demonstrate the architectural feasibility and system-level behavior of distributed event-driven orchestration under resource-aware multicluster deployment.

To further analyze how multicluster configurations influence resource utilization during event processing, an additional experiment was conducted in which the number of clusters was increased from one to three while measuring CPU usage. Each experiment was performed on clusters with identical specifications (4 vCPU, 8 GB Memory, and 74 GB storage), and between 150 and 500 concurrent HTTP events (Webhook requests) were generated to observe the average CPU utilization during the event-processing interval.

The average CPU utilization across C clusters when processing E concurrent events is defined as follows:

$$\bar{U}\left(C,E\right)=\frac{1}{C}\sum_{i=1}^{C}{\mathrm{CPU}}_{i}\left(E\right)$$

- $\bar{U}\left(C,E\right)$: The average CPU utilization when the number of clusters is *C*.
- ${\mathrm{CPU}}_{i}\left(E\right)$: CPU utilization of cluster *i* when processing concurrent events *E*.

**Figure 13:**
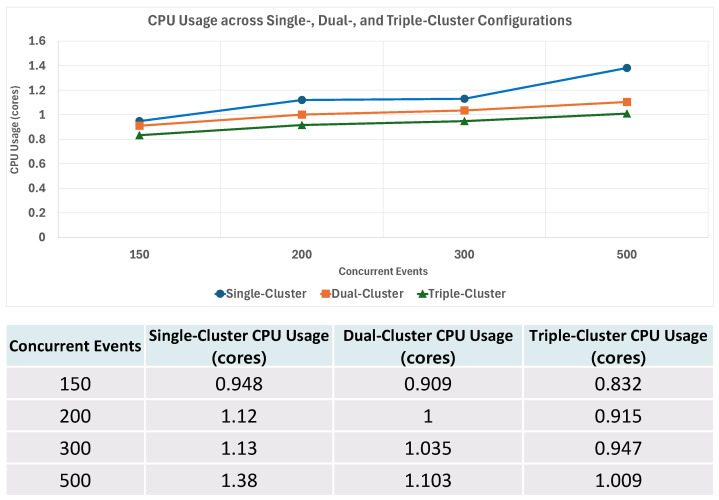
Comparison of CPU utilization across single-, dual-, and triple-cluster configurations under increasing concurrent events.

Figure 13 compares CPU utilization across single-, dual-, and triple-cluster configurations under increasing concurrent events.

When generating between 150 and 500 concurrent events, CPU utilization in the single-cluster configuration increased more rapidly as the event volume grew, approaching resource saturation under the 500-event workload. In contrast, the dual- and triple-cluster configurations exhibited more gradual increases in average per-cluster CPU utilization, as the event load was distributed across multiple clusters.

It is important to clarify that the reported values represent the average per-cluster CPU utilization, defined in Equation (3), rather than the total aggregated CPU usage across all clusters. Since the total available compute capacity increases with the number of clusters, the observed stabilization in per-cluster CPU usage reflects both workload distribution and increased aggregate resources. A capacity-matched comparison would be required to isolate the specific contribution of dynamic placement, which is left for future work.

To further analyze how the number of clusters influences overall resource usage, we also examined the pattern of memory consumption across different cluster configurations. For this purpose, the average memory usage of each cluster *i* during the event-processing period was defined as follows:

$$\bar{M}\left(C,E\right)=\frac{1}{C}\sum_{i=1}^{C}{\mathrm{Mem}}_{i}\left(E\right)$$

- $\bar{M}\left(C,E\right)$: The average memory utilization measured when the number of clusters is *C*.
- ${\mathrm{Mem}}_{i}\left(E\right)$: Memory usage of cluster *i* when *E* concurrent events occur.

**Figure 14:**
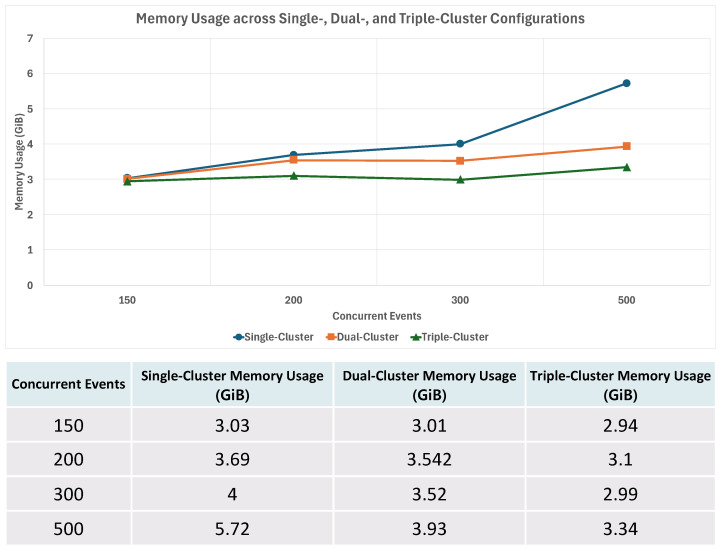
Comparison of memory utilization across single-, dual-, and triple-cluster configurations under increasing concurrent events.

Figure 14 compares memory utilization across single-, dual-, and triple-cluster configurations under increasing concurrent events.

In the single-cluster configuration, all Workflow Pods and event-processing resources were concentrated within a single cluster. As the number of concurrent events increased, memory usage accumulated rapidly due to the simultaneous creation and execution of Pods in the same resource domain. Under high-load conditions, this led to a steep increase in memory consumption and elevated resource pressure within the cluster.

In contrast, in multicluster configurations, pipeline components were distributed across multiple clusters. As a result, memory usage was spread across separate resource domains, leading to lower average per-cluster memory pressure as workload increased, while total system-wide memory usage naturally grew with the number of concurrent events, no individual cluster experienced abrupt memory saturation under the tested conditions.

It is important to interpret these results in the context of aggregate system capacity. Increasing the number of clusters proportionally increases the total available memory resources. Therefore, the observed stabilization in per-cluster memory usage reflects both workload distribution and increased aggregate capacity. Overall, these findings illustrate the workload distribution behavior of the proposed architecture under distributed deployment, rather than establishing strict causal superiority of the orchestration mechanism alone.

### 5.4. Limitations and Scope of Evaluation

The evaluation presented in this study focuses on demonstrating the end-to-end feasibility and performance behavior of the proposed multicluster orchestration architecture under increasing event workloads. We acknowledge that the experimental comparison between single-cluster and multicluster configurations does not include a capacity-matched baseline, such as a vertically scaled single cluster with equivalent total resources. As a result, improvements in stability and success rate may be influenced both by distributed orchestration and by increased aggregate system capacity.

Furthermore, the workload was generated through controlled HTTP webhook injections to emulate smart-city-inspired scenarios rather than using real-world IoT traces. Therefore, the results should be interpreted as an experimental validation of architectural scalability trends rather than a definitive real-world deployment study. Future work will include capacity-isolated comparisons, burst-pattern modeling, and real-world workload traces to further strengthen causal attribution and external validity.

## 6. Conclusions

In this study, we designed and implemented a distributed event-driven Function as a Service (FaaS) orchestration platform that overcomes the scalability and reliability limitations of traditional single-cluster serverless environments. The proposed system integrates Argo Events, Argo Workflows, and Knative Serving on top of a Karmada-based multicluster management framework, enabling the automatic generation and management of EventSource, Sensor, WorkflowTemplate, Knative Service, and PropagationPolicy resources through a single user-defined Custom Resource. Furthermore, a Prometheus based PlacementDecision Controller continuously collects and normalizes CPU and memory utilization metrics from each cluster, computes resource scores, and dynamically assigns the event processing pipeline to the most suitable cluster. Experimental results indicate that the proposed multicluster orchestration approach can improve load distribution and maintain higher workflow success rates under increasing event loads compared to the evaluated single-cluster baseline.

For future work, we plan to reduce inter cluster communication latency by adopting Cilium Global Service- or Istio Ambient Mesh-based communication optimization, and to improve placement accuracy using AI-driven scoring models. We also aim to strengthen multicluster event delivery reliability and fault tolerance by incorporating message replication (Mirror/Source) and Leaf-node mechanisms into the distributed event bus. Overall, this research presents an architectural direction that significantly improves automation, scalability, and resilience in event-driven serverless systems deployed across multicluster environments, and it offers strong potential for IoT, smart city, and real-time AI applications that require robust distributed event processing.

## Figures and Tables

**Table 1:** Environment and software configurations used in the multicluster testbed.

Component	Specification/Version
OpenStack Instance × 4 4	Compute Optimized <m1.large.flavor>
Operating System	Ubuntu 20.04 LTS
Kubernetes	v1.29.15
Karmada	v1.14
Argo Events	v1.9.4
Argo Workflows	v3.5.4
Knative Serving	v1.16.1

## Data Availability

No new data were created or analyzed in this study. Data sharing is not applicable to this article.
